# An Exploratory Study on Determining and Modeling the Creep Behavior of an Acrylic Pressure-Sensitive Adhesive

**DOI:** 10.3390/ma16052029

**Published:** 2023-03-01

**Authors:** Beatriz D. Simões, Élio M. D. Fernandes, Eduardo A. S. Marques, Ricardo J. C. Carbas, Steven Maul, Patrick Stihler, Philipp Weißgraeber, Lucas F. M. da Silva

**Affiliations:** 1 Institute of Science and Innovation in Mechanical and Industrial Engineering (INEGI), Rua Dr. Roberto Frias, 4200-465 Porto, Portugal; 2 Departamento de Engenharia Mecânica, Faculdade de Engenharia, Universidade do Porto, Rua Dr. Roberto Frias, 4200-465 Porto, Portugal; 3 Robert Bosch GmbH, Corporate Research and Advance Engineering, 71272 Renningen, Germany; 4 Faculty of Mechanical Engineering and Marine Technology, University of Rostock, 18059 Rostock, Germany

**Keywords:** pressure-sensitive adhesives, creep modeling, adhesive bonding

## Abstract

In the present paper, an exploratory study on the creep behavior of a pressure sensitive adhesive (PSA) is performed. After the determination of the quasi-static behavior of the adhesive for bulk specimens and single lap joints (SLJ), SLJs were subjected to creep tests at 80%, 60%, and 30% of their respective failure load. It was verified that the durability of the joints increases under static creep conditions as the load level decreases, with the second phase of the creep curve becoming more pronounced, where the strain rate is close to zero. In addition, cyclic creep tests were performed for the 30% load level at a frequency of 0.04 Hz. Finally, an analytical model was applied to the experimental results in order to reproduce the values obtained for both static and cyclic tests. The model was found to be effective, reproducing the three phases of the curves which allowed for the characterization of the full creep curve, something not commonly found in the literature, especially for PSAs.

## 1. Introduction

In recent years, the use of pressure-sensitive adhesives (PSAs) has vastly expanded, particularly in the electronics sector [1,2]. These adhesives possess characteristics that are attractive for industrial applications, as they facilitate bonding processes due to the initial high adhesive force and allow for the joining of two parts without the necessity of fixation. This enables faster production cycles and easier handling. Furthermore, this has been accompanied by a constant research effort, seeking to improve their long-term reliability and mechanical behavior [3]. These materials, which generally exhibit viscoelastic properties, are characterized as being able to establish a strong bond between solid substrates, such as metal and non-metal surfaces [4], only through the application of slight pressure, immediately tacking the surface once there is sufficient contact [5,6,7]. This means that, even though they can conform easily to the surface being bonded and achieve a good level of contact, they have enough flow resistance throughout the separation and debonding processes [2]. Besides having tack properties, adhesive bonding when producing a joint configuration occurs without the need for any kind of activation, such as the application of heat, the presence of water, or the release of a solvent [5,8]. The major distinction between PSAs and other types of adhesives is that, in the former, the adhesive surface’s characteristics remain constant before and after application [9]. PSA materials are divided into acrylic [10], rubber [6], silicone [9], and urethane-based [11] categories, according to the type of polymer incorporated [12]. Among the various options, PSAs belonging to the acrylic class have several advantages compared to the others. These include excellent transparency, adhesive properties, photo-curing properties, weather resistance, heat resistance, and aging resistance [12]. Additionally, these adhesives are the most widely used in industrial applications, since they contain monomers that can be tailored to specific functionalities [13]. These applications include splicing tapes, graphic films, display products, and medical products [2,13,14].

When polymeric materials are subjected to a constant load below their maximum strength for long periods of time, they are known to be susceptible to the creep phenomenon [15,16,17]. Polymer creep is caused by viscoelastic deformation, which is a combination of elastic deformation and viscous flow [18]. In the particular case of adhesives, this phenomenon can occur at low temperatures and low stress levels [15,19], and its behavior depends on the applied load and service temperature [15,20]. Creep is defined as the time-dependent permanent deformation of a material caused by the application of constant structural stress for an extended period of time at a constant temperature [21]. Adhesives subjected to this phenomenon exhibit deformation that can be characterized by three different stages of creep—primary, secondary, and tertiary creep [1,22,23]. The first stage starts with a short transient period before secondary creep is reached, which is considered stationary, and the dominant creep mode [23]. In this part of the phenomenon, it is assumed that an equilibrium is reached between softening and hardening of the adhesive, which gives rise to a stable strain rate. It is in the third phase that a rapid increase in strain rate is observed, which accelerates until failure [18,23]. Extensive research has been made on creep during the last decades, which allows for a better understanding of the creep behavior of various materials. Research on creep has been carried out through experimental and computational approaches [24] and has been expanded to the particular case of the creep phenomenon in adhesives [17,18,23,24,25,26]. However, an in-depth study of the creep behavior of PSAs is still lacking in the literature [1].

For example, Geiss and Vogt [27] applied standard tests, used to characterize structural adhesives, to study semi-structural bonds using PSAs and subjected to common aging conditions. In this study, a single cantilever wedge test was used to evaluate the durability of interfacial adhesion. The adhesion of PSAs to glass was found to be strongly dependent on the moisture level, although the authors have also concluded that the performance could be significantly improved by applying a primer prior to the bonding of the two materials. Additionally, Townsend et al. [22] characterized the creep behavior of a high-performance acrylic PSA as an alternative joining method for structural glazing. For comparison purposes, creep tests were also performed on three different one- and two-part silicones. In comparison with the reference materials, the failure time parameter of PSA proved to be more sensitive to the level of the applied load, and the failure time of the silicones, on average, was higher. Thus, for the studied PSA considered for structural applications, a larger joint width was found to be required compared to the silicone sealant materials. Despite these results, if the load and strain rate were increased, the difference between the two adhesives was found to decrease. Additionally, it was verified that the presence of defects in silicones has a greater impact on their creep life than in acrylic PSAs. In another study, Ernault et al. [28] studied the creep behavior in single-lap joints (SLJ) of two hot melt PSAs including a semicrystalline adhesive and an amorphous adhesive. Glass and stainless-steel substrates were used, which demonstrated that the viscoelastic response of these adhesives is highly sensitive to substrate properties. The observed behavior could be explained by the different wettability values that the two materials demonstrated, with better adhesion being achieved for the glass substrates. For stiff adhesives, joint viscoelasticity has been shown in the literature to follow the same behavior as the bulk material. However, in the case of these highly flexible adhesives, this does not apply. Additionally, the authors noted that the performance of the two adhesives under creep was very different. Although the semicrystalline PSA withstood a higher load, when comparing shear strain as a function of time, for similar strains, the same adhesive showed a better initial performance but presented an early catastrophic failure. These results can be explained due to its viscoplastic behavior accompanied by the necking phenomenon.

Although the definition of creep is intrinsically linked to inelastic deformation caused by the application of constant structural stress, it has already been demonstrated in the literature that fatigue failure at low frequencies is controlled by creep phenomena [29]. The study of inelastic deformation when materials are subjected to cyclic loading has been widely developed over the past decades [30,31,32,33,34]; however, studies devoted to adhesive joints have been more limited [29,35,36], especially for the specific case of PSAs [37].

Several studies have been conducted with the aim of creating models able to predict the creep behavior of polymers [38,39,40] since knowledge of this behavior is required for various applications in different industries [25]. When modeling creep phenomena, in any material, empirical models and rheological models can be used [41]. However, due to the fact that most research focuses on predicting the strains that result from the creep process, rather than studying the failure process in detail, the use of models that simulate the complete creep curve is very limited, that is, models that predict the behavior at the primary, secondary, and tertiary creep stages [23].

Huang et al. [42] addressed the uniaxial tensile creep response of acrylic-based PSAs where a multistage creep response was observed. This phenomenon occurs when joints composed of rigid substrates bonded by a very ductile single-layered PSA are subjected to tension loading. This behavior is justified by the fact that during testing there are competing softening and hardening mechanisms, namely cavitation in the former and fibrillation in the latter. Additionally, the interaction between them is dependent on the properties of the adhesive, as well as on the properties of the substrates. Taking this behavior into consideration, the authors presented a predictive mechanistic creep model that was based on the phenomena of cavitation, the growth of cavities, both interfacial and bulk fibrillation, and mechanical locking. The same model was found to be suitable to reproduce the various stages presented by the experimental results, as well as able to clarify the effects of PSAs and substrate properties on the joint creep response. It is important to note that the model developed by the authors cannot be applied to any type of load, but only to joints under uniaxial tensile loading. Duan et al. [23] developed a unified phenomenological model, considering uniaxial compression creep testing, in polymer-bonded composite materials. This model allowed for the prediction of creep behavior at all stages of the curve for different load levels and temperatures. The results obtained were compared with a reference model, for which it was possible to reduce the errors by approximately 28%. Additionally, they concluded that the Larson-Miller parameter can be used to predict the time to failure in these materials and that, together with the developed model, it can be used to predict the creep behavior, even in the absence of test data. However, due to its phenomenological nature, the proposed model can predict strain rate and rupture time but cannot explain in detail the creep mechanisms in these materials.

The aim of the present work was to perform an exploratory study, seeking to experimentally characterize and model the creep behavior of PSAs subjected to shear loads. An acrylic PSA was chosen for the testing campaign, and its behavior was evaluated both in terms of static and cyclic creep. The experimentally obtained data were then used to feed a model based on that developed by Duan et al. [23], allowing us to understand its suitability for modeling the creep behavior of PSAs. Additionally, the same model was used to reproduce a cyclic creep curve, using the displacements obtained by digital image correlation (DIC) analysis.

## 2. Experimental Details

### 2.1. Materials

The acrylic PSA used in the present work is a high-performance acrylic adhesive, with good long-term strength and temperature stability. It can be used in industrial applications due to its capacity to bond different materials in a variety of adverse environments. The manufacturer’s datasheet indicates a Young’s modulus of $4.5\times 10^{-1}$ MPa and a Poisson’s ratio of 0.499 for this adhesive.

The metallic adherends used in the tested SLJs were manufactured from an aluminum alloy of the 6082-T6 series. The 2 mm thick sheets have a Young’s modulus of 70 GPa and a Poisson’s ratio of 0.33.

### 2.2. Specimen Manufacturing

#### 2.2.1. Bulk Specimens

**Figure 1:**
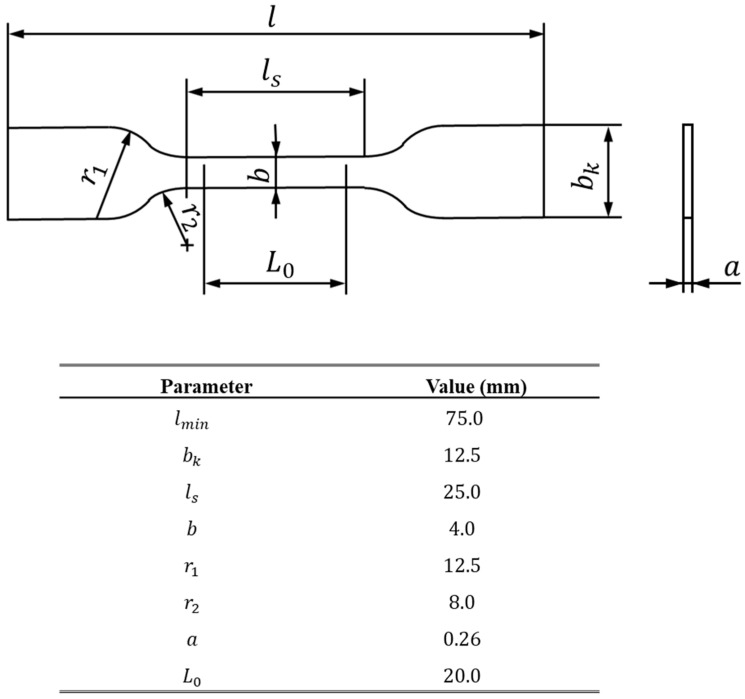
Bulk specimen’s geometry (**top**) and the respective values in mm (**bottom**).

Dogbone bulk specimens were used to characterize the tensile behavior of the acrylic PSA, with a geometry based on the German standard DIN 53504 [43] represented in Figure 1. However, given the nature of the used adhesive—supplied in sheets with a preset thickness—the thickness of the specimen deviated from the value specified by the standard, corresponding to 0.26 mm.

Considering that the used acrylic PSA was supplied in sheets, it was necessary to cut the material to match the final geometry. To this end, a CAD model was created and, finally, the specimens were cut out using the 3D printed part as a precise cutting guide.

After the desired geometry was obtained, aluminum tabs were added at the end of the specimens. This step was necessary so that the specimens could be attached to the gripping system of a universal testing machine.

#### 2.2.2. SLJ Specimens

**Figure 2:**
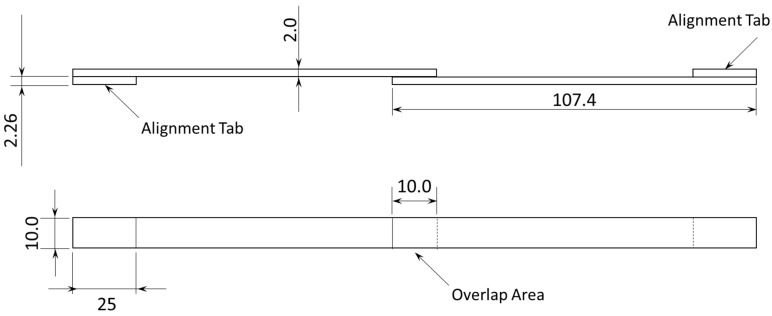
SLJ specimen’s geometry in mm.

SLJs were used for three different testing procedures, namely quasi-static tests, static creep tests, and cyclic creep tests. The first had the function of defining the maximum static load to which this adhesive could support in a joint configuration. The second was employed to determine the behavior of the adhesive to static creep under various load levels, that is, the percentage of the maximum transmissible static load determined in the quasi-static SLJ tests. Finally, the third testing procedure served as an initial study of the behavior of this adhesive, when subjected to the cyclic creep phenomenon, only for one load level. Figure 2 shows the specimens’ geometry used to perform the aforementioned tests.

**Figure 3:**
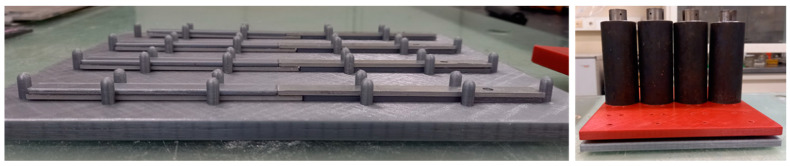
Joints after assembly (**left**) and the final result with the weights placed (**right**).

To accurately obtain 10 × 10 mm squares of adhesive, scalpels were used to cut the desired shapes. Then, after cleaning the surface with acetone, the material was placed on the aluminum adherends. A specially designed mold was used for the joints in consideration, ensuring both the perfect alignment of the two adherends and a 10 mm overlap. After the joints were assembled, 2 kg weights were placed in the center of each joint for 48 h so as to promote good adhesion between all parts. Figure 3 on the left shows a set of four joints after their assembly, and on the right, the result is shown after the weights were placed in their respective locations.

Finally, to allow for further DIC analysis in the case of cyclic testing, the specimens that were to be subjected to this type of loading were coated with white matte paint and speckled with a black ink dot pattern.

### 2.3. Testing Setup

This section describes the test procedures used in this work, namely bulk tensile, quasi-static, static creep, and cyclic creep testing. For all tests, at least three specimens were tested in order to ensure the repeatability of the results.

#### 2.3.1. Bulk Tensile Testing

The bulk tensile tests were performed on a twin-column tensile tester Mecmesin^®^ MultiTest-10i (Physical Properties Testers, Halifax, UK) equipped with a 10 N load cell, and the tensile-displacement curves were recorded for each test until the failure of the specimen. All specimens’ dimensions were measured before each test, ensuring the accuracy of the tensile stress measurements. All tests were performed at a constant crosshead speed of 1 mm/min in order to determine the tensile strength of the adhesive. The specimens were attached to the machine resorting to clamps that held both ends of the specimen through aluminum tabs that were previously added.

#### 2.3.2. Quasi-Static Testing

For the quasi-static condition in SLJs, an INSTRON^®^ 3367 (Illinois Tool Works, Hopkinton, MA, USA) universal testing machine was used, equipped with a 30 kN load cell. All tests were performed at a constant crosshead speed of 1 mm/min, aiming to determine the strength of the adhesive when used in an SLJ. The specimens were fixed to the machine using clamps that held both ends of the joints, whereupon the aluminum tabs ensured the alignment of the joint under load.

#### 2.3.3. Static Creep Testing

**Figure 4:**
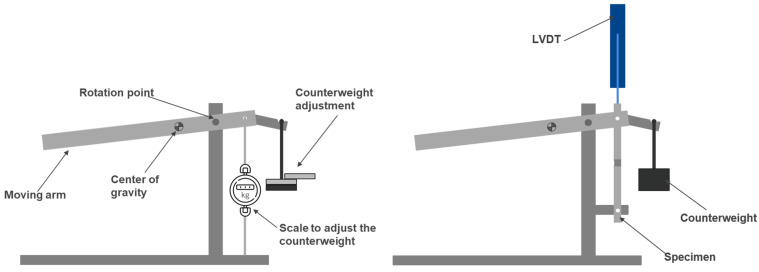
Static creep machine representative scheme: counterweight adjustment (**left**) and unit ready to test (**right**).

The static creep testing was performed resorting to a modified G.U.N.T.^®^ WP 600 (G.U.N.T. Gerätebau GmbH, Barsbüttel, Germany) experimental unit, schematically represented in Figure 4. After determining the strength of SLJs under quasi-static loading, it was necessary to adjust the weight submitted to the joint in the creep test. A load cell was positioned where the joint would later be inserted, and the weights were adjusted according to the desired load level—80%, 60%, or 30% with respect to the failure load. Due to the self-weight of the moving arm, various sets of weights were applied as counterweights until the desired condition was achieved, as shown in Figure 4 on the left.

Having defined the weights to be used for the different conditions, an LVDT (Linear Variable Differential Transformer) was positioned in line with the joint end to exactly measure the displacement during creep testing. Figure 4 (right) demonstrates a schematic representation of the experimental unit ready to test a joint, equipped with the appropriate counterweight, as well as the displacement sensor.

#### 2.3.4. Cyclic Creep Testing

**Figure 5:**
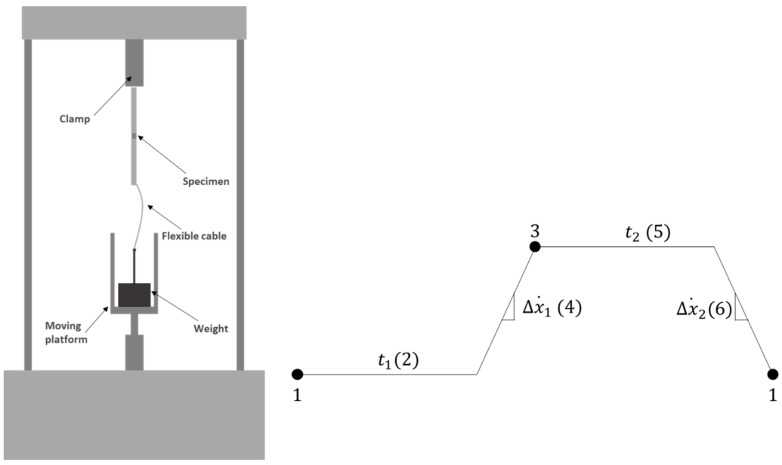
Servo-hydraulic machine for cyclic creep testing representative scheme (**left**) and schematic stages for wave generation (**right**).

For the cyclic creep testing, an INSTRON^®^ 8801 (Illinois Tool Works, Hopkinton, MA, USA) servo-hydraulic testing machine was used, and the specimens were tested in displacement control, using the waveform generator provided by the manufacturer’s software. This feature allowed for the generation of a trapezoidal wave by defining several characteristic points. Thus, the following points were determined: (1) initial position, (2) initial position duration, (3) final position, (4) velocity of displacement to the final point, (5) final position duration, and (6) velocity of displacement to the initial point, as shown in Figure 5 (right). By defining the displacement velocities between the two extreme positions, as well as the duration of this same state, it was possible to determine a constant test frequency of 0.04 Hz for all tests performed. The specimens were fixed using clamps holding one end of the joint. On the other side, a flexible cable was used to connect the joint to the weight. To ensure that the joint was in both states of cyclic loading—loaded and unloaded, a platform was placed on the machine actuator. In this way, as it performed upward and downward movements, defined by the trapezoidal wave, the joint moved from the loaded to the unloaded state consecutively until failure was reached. The use of a cable instead of a fixed linkage to support the weight enables the desired load level to be achieved in a gradual manner and allows for the specimen to be unloaded when the weight is supported by the moving platform installed in the servo-hydraulic testing machine. Figure 5 (left) depicts a schematic representation of the used apparatus.

In order to guarantee that a correct 2D DIC analysis could subsequently be performed, it was necessary to synchronize the start and end of the tests, with the start and end of the images obtained. Additionally, it was necessary to make sure that at each cycle, a picture at the maximum and minimum displacement stages would be taken. To do this, the tests were synchronized, and the photo interval was set using a Nikon D5300 (Tokyo, Japan) digital camera with a Nikon AF-P NIKKOR 18–55mm f/3 lens attached.

### 2.4. DIC Analysis

**Figure 6:**
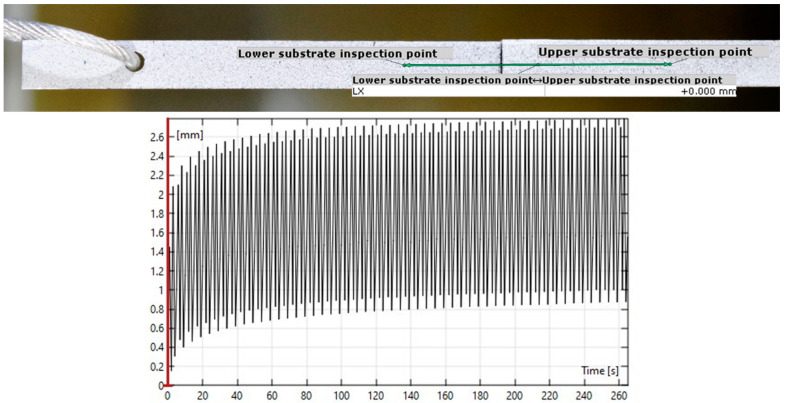
DIC analysis of a cyclic creep test: marked inspection points (**top**) and the resulting plot of the displacement through the test (**bottom**).

The captured images were analyzed using the GOM Correlate software (2019, Carl-Zeiss-Stiftung, Stuttgart, Germany) in order to acquire the displacement level experienced in the joint throughout the test. Considering that the elastic deformation of the adherends will be negligible, compared to the deformation of the adhesive, given the value of the loads in question, it was assumed that the displacement of the aluminum substrates could be considered a rigid body movement. Thus, the relative displacement between two points on the substrates would be equivalent to the displacement observed in the adhesive itself. Figure 6 shows an example of a DIC analysis of a cyclic creep test, where the two points used for the displacement measurement can be seen, as well as the plot of the relative displacements recorded during the test.

## 3. Analytical Creep Model

In the present work, the model proposed for polymer-bonded composite materials by Duan et al. [23], named the creep strain rate model, was considered to study the accuracy of the model for the characterization of all three creep phases for an acrylic PSA confined in an SLJ. In [23], Equation (1) was proposed to describe the logarithmic transformed creep strain data.

(1) $$\ln\frac{\partial \varepsilon }{\partial t}=\frac{a}{{\left(t-t_{ini}\right)}^{b}\times {\left(t_{rup}-t\right)}^{c}}$$

where a, b, and c are curve-fitting parameters, and $t_{ini}$ and $t_{rup}$ are the values of initial and rupture time of the considered data, respectively. The parameter a is related to the slope of the secondary creep phase, which is almost linear, whilst b and c are related to the transition between the creep phases, with b being related to transition from primary to secondary creep and c from secondary to tertiary.

From (1), the strain rate is obtained:

(2) $$\dot{\varepsilon }\left(t\right)=\frac{\partial \varepsilon }{\partial t}=\exp\left(\frac{a}{{\left(t-t_{ini}\right)}^{b}\times {\left(t_{rup}-t\right)}^{c}}\right)\;$$

Integration of the strain rate yields the strain as a function of time:

(3) $$\varepsilon \left(t\right)=\int_{t_{ini}}^{t}\dot{\varepsilon }\left(\tau \right)\;d\tau +\varepsilon_{0}\;$$

where $\varepsilon_{0}$ represents the initial elastic deformation.

The proposed model was applied using experimental data together with the aforementioned equations. Since the results obtained using the LVDT represents the joint displacement, these values were first converted into strains, and only then the strain rate was calculated throughout the test. The curve fitting was performed using Equation (2) since the results present less error than using Equation (1) directly. Such evidence was also found in the work developed in [23].

To determine the average error, the local error was calculated between each point of the experimental data and the respective point in the approximation curve, as shown in Equation (4):

(4) $$Average\;\;error=\frac{1}{N}\left(\;\overset{N}{\underset{i=1}{\sum }}\frac{│\varepsilon_{i}^{curve}-\varepsilon_{i}^{experimental}│}{\varepsilon_{i}^{experimental}}\right),\;\;N=No.\;experimental\;points\;$$

### Cyclic Creep Model

**Figure 7:**
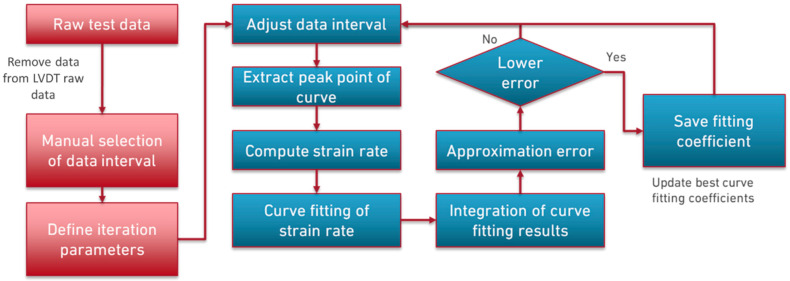
Flowchart of the cyclic creep model.

The model used to characterize the adhesive behavior when subjected to cyclic creep differs from that used for the static creep case only in the way the displacements were processed. In the case of this type of loading, it was defined that the maximum displacements in each cycle would be the most interesting for the analysis in question. Thus, after defining the relevant interval for the obtained data, a selection of the maximum displacement points extracted from the DIC measurements for each cycle was included. Finally, the model followed in an identical manner to the case of static loading. Figure 7 depicts the developed model for the cyclic creep condition.

## 4. Results and Discussion

### 4.1. Bulk Tensile and Quasi-Static Tests

**Figure 8:**
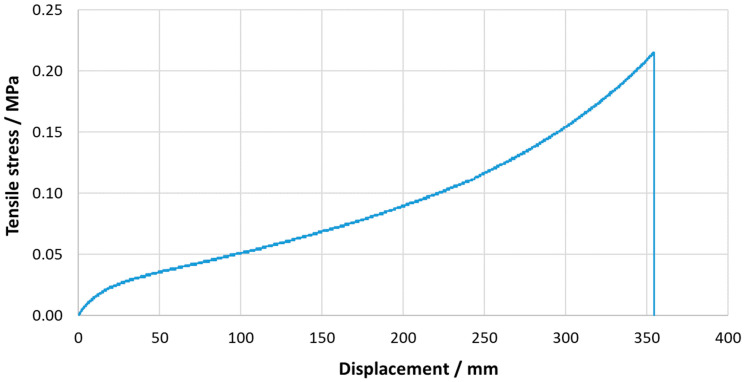
Tensile stress–displacement representative curve for the acrylic PSA bulk specimens.

Bulk tensile tests were performed to determine the ultimate stress of the adhesive used in this work. Figure 8 represents the values obtained for loading at 1 mm/min, until the total failure of the specimen, which allowed us to find its tensile strength as well as its critical displacement. A good level of repeatability was observed, with a maximum stress value of 0.21±0.01 MPa and a critical displacement of 353.70±1.23 mm.

**Figure 9:**
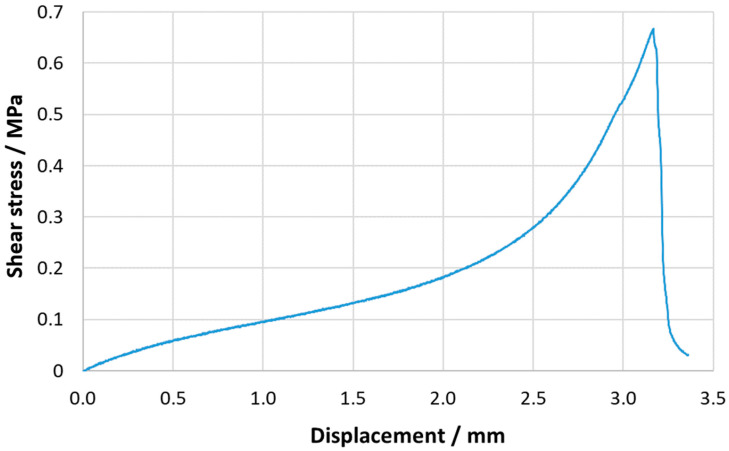
Shear stress–displacement representative curve for the acrylic PSA SLJ quasi-static tests.

Figure 9 shows the shear stress–displacement representative curve obtained for the quasi-static tests of the SLJs, from which their shear strength as well as their critical displacement can be evaluated. Good repeatability of the results was observed, with an average shear strength value of 0.64±0.06 MPa.

### 4.2. Static Creep

**Figure 10:**
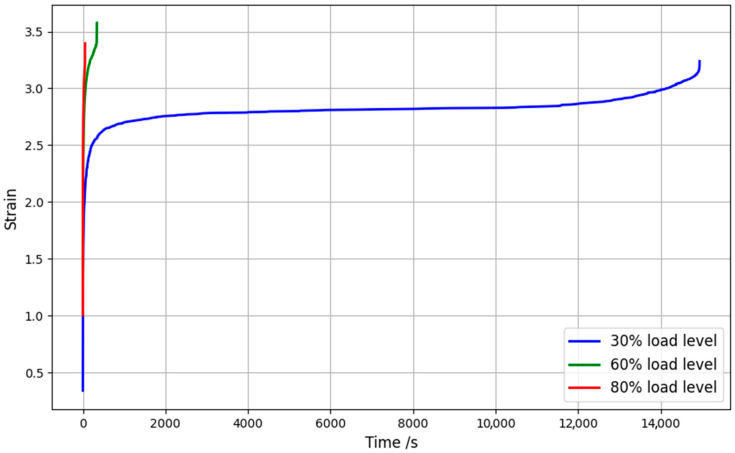

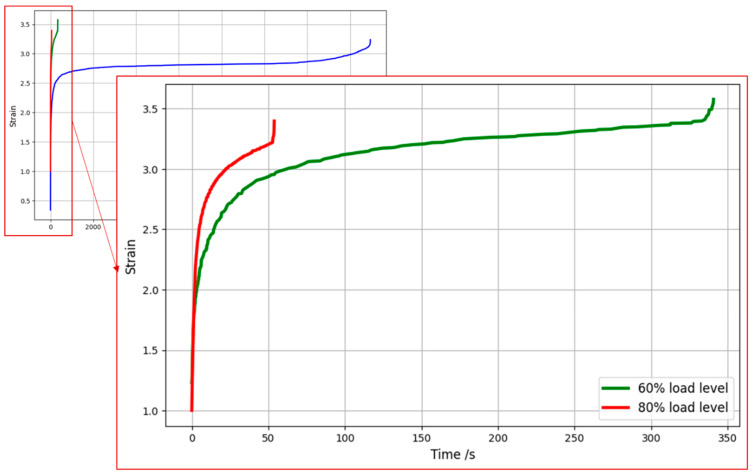
Experimental static creep representative curves for 30%, 60%, and 80% load level: three levels (**top**) and higher levels in detail (**bottom**).

Static creep tests were performed for load levels of 30%, 60%, and 80% of the maximum failure load determined in the SLJs. Figure 10 shows the representative curves for each load level where it can be seen that, as expected, the closer the load level is to the maximum stress value obtained in the tests in quasi-static conditions, the smaller the rupture time. When comparing the three levels on the same graph, the secondary phase of the curves is more developed for the 30% level, since the joint is subject to lower stresses. Thus, for the 80% and 60% load levels, the tertiary phase, where damage occurs and the strain rate increases significantly, takes place very close to the beginning of the test.

**Figure 11:**
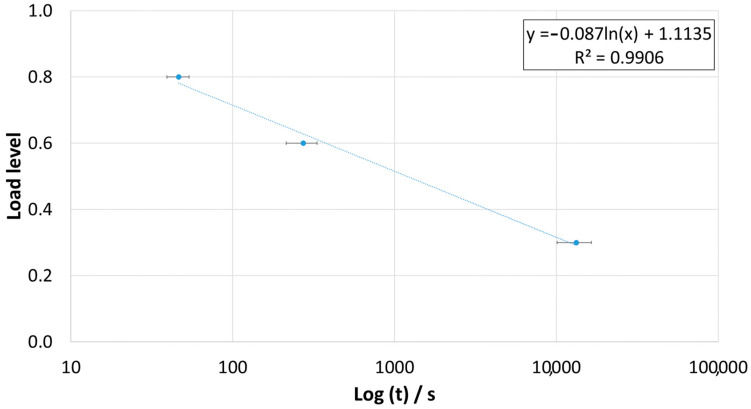
Time to failure (s) of the static creep tests as a function of the load level.

Analyzing the trend for the failure time of this adhesive as a function of the load level, it can be seen that if the times are plotted on a logarithmic scale, then the values follow a linear trend. Figure 11 shows the agreement between the experimentally obtained points and the trend line used to correlate the data.

**Figure 12:**
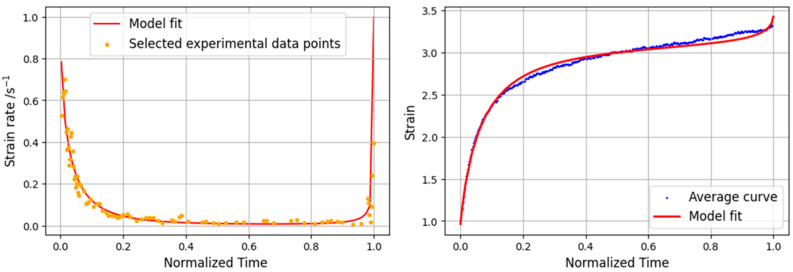
Analytical and experimental strain rate curve (**left**) and static creep curve (**right**) for 80% load level.

The data obtained from the tests were used in the previously described creep strain rate model. In order to be applied to the average curves obtained for each load level, these were first normalized. After normalization, the curves of the experimental results were filtered so that they contained the same number of points. Thus, the average of the values of the curves for each point was determined. Figure 12 depicts the results obtained for the 80% load level. Since this level is very close to the maximum joint stress, the portion of the strain rate curve corresponding to the secondary phase of the creep curve, where the strain rate is nearly zero, corresponds to just a small part of the overall creep curve. The model shows a good correlation with the experimental data, especially in the primary and tertiary zone.

**Figure 13:**
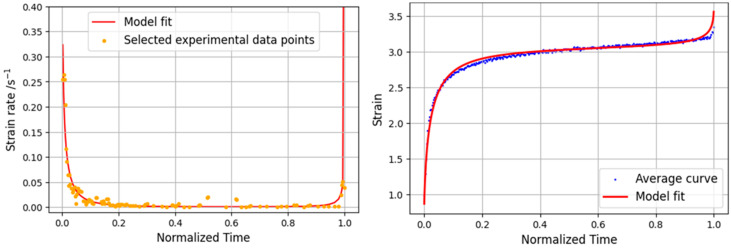
Analytical and experimental strain rate curve (**left**) and static creep curve (**right**) for 60% load level.

For the 60% case, as can be seen in Figure 13 (left), the strain rate values are generally lower than those corresponding to the 80% level. Additionally, the stable zone corresponding to the secondary creep is now larger, which is in line with the evolution of the curves. For both the strain rate and the creep curve, the model presents good overall agreement for all phases of the curve, as shown in Figure 13 (right).

**Figure 14:**
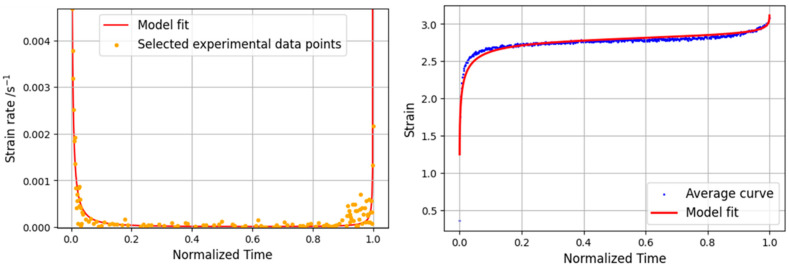
Analytical and experimental strain rate curve (**left**) and static creep curve (**right**) for 30% load level.

For the 30% load level, the reduction in the order of magnitude of the strain rate values is evident. This behavior is consistent with a duration more than 50 times longer than the other load levels, resulting in a progressive evolution of the creep curve, with a very large secondary phase. Due to the very high value of the initial slope of the experimental curve, the analytical model encountered some difficulties in approaching the initial value of the strain; although, in the rest of the curve, the correlation is very high. These problems occurred because the model resorts to an exponential equation, and when the initial slope is too high, the transition from primary to secondary creep is difficult to fit. Figure 14 presents both strain rate evolution and creep behavior of the lowest load level.

After generating the analytical curves fitted with the experimental results, Equation (4) was used to determine the value of the average error, which is calculated by comparing the analytical curve against the average experimental curve. Thus, it was verified that the curves obtained by the model presented a satisfactory level of correlation, with values never exceeding 1.5%. This means that, although the experimental results may present higher values of dispersion, the model accurately approximates the resulting average curves in all its phases.

### 4.3. Cyclic Creep

**Figure 15:**
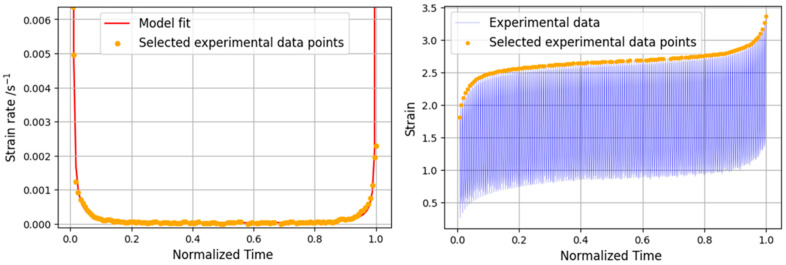
Strain rate calculated for cyclic creep test (**left**) and points extracted from the cyclic strain-time curve to use in the model (**right**).

So far, there is no known analytical model for acrylic PSA materials confined in joints that characterizes the cyclic creep behavior for all phases of the creep curve. Thus, with this work, it was also intended to initiate a study about the feasibility of using the model proposed in the present investigation to do so. This first approach consisted in submitting the joint at a load level of 30% with a cyclic loading of 0.04 Hz and then analyzing its displacements and strains. As the current goal of the work is to understand if the model can be applied to this case, the result is displayed in a normalized curve in order to represent the average of the tests performed. Figure 15 in the left depicts the strain rate results obtained from the cyclic tests, as well as the points used for the approximation curve. On the right, the cyclic strain–time curve is shown, as well as the points extracted from the curve to be used in the model.

Regarding strain rate, the curve has a typical appearance, similar to what is obtained in the static analysis. It starts with higher values that decrease abruptly and correspond to the primary phase of the creep curve. This is followed by a stable zone with a value of almost zero, corresponding to the secondary phase, with a practically linear evolution of the strain. Finally, the value of the strain rate rises rapidly again, which corresponds to the tertiary phase, where adhesive damage occurs, prior to joint failure. In the cyclic strain curve, it is possible to clearly identify the cycles to which the joint was subjected during the test. Choosing the maximum displacement points and, consequently, the highest strains, for each cycle, there is a clear tendency that can be categorized as identical to that observed in the creep phenomenon, with the three phases well-defined. This result is compatible with what is observed in the evolution of the strain rate.

**Figure 16:**
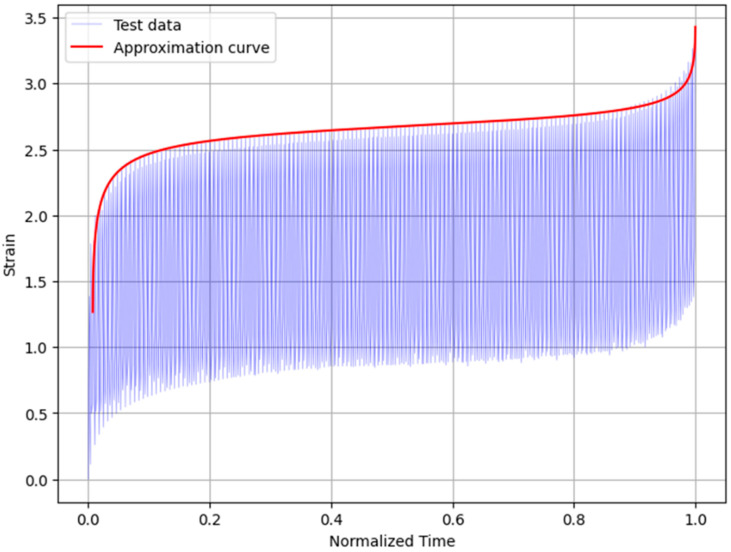
Cyclic strain–time curve with the approximation curve generated using the model.

Figure 16 shows the cyclic strain–time curve, as well as the approximation curve generated by the model used. From what can be observed, the model seems to present a good level of agreement with the maximum strains reached during the test. All creep phases are well approximated, and the curve adequately represents the creep behavior of these materials. Thus, this preliminary result seems to provide a reasonably accurate methodology to model the cyclic creep phenomenon in PSAs.

## 5. Conclusions

In this work, the creep behavior of an acrylic PSA was modeled using a creep strain rate approach, both for static and cyclic creep conditions. To support this analysis, bulk testing of the adhesive and SLJ testing was carried out under quasi-static conditions, using an adapted testing apparatus, based on a fixed load. Creep displacement was tracked using an LVDT. Cyclic creep testing was also carried out using a novel testing procedure, whereupon a servo-hydraulic testing machine was programmed to load and unload a joint connected to a weight. By removing support to this weight, the joint could be creep-loaded in a cyclic manner. Before creep testing took place, quasi-static joint strength was established. Static creep tests were then performed for 80%, 60%, and 30% of this value, and the analytical model, first developed by Duan et al. [23], was used to model performance under all these levels. Finally, cyclic creep tests were performed for a load level of 30%, and for this condition, the model was also applied. To conclude:

- The static creep results are in line with what was expected, that is, the smaller the load, the higher the rupture time;
- For the 60% and 80% load levels, the stable secondary creep phase is not very prominent, and there is a more pronounced presence of the higher strain rate phases;
- For the case of the 30% load level, the tests lasted more than 50 times longer than the other load levels, with a very pronounced stable phase corresponding to the secondary creep phase. This behavior is in accordance with a more favorable stress state with less load level, presenting a slower progression of strains;
- The creep strain rate model proved to be able to correctly characterize the creep behavior of the various load levels tested;
- Although some details of the experimental curves may be more difficult to fully match, all three phases of the curve have been satisfactorily modeled, with a special focus on the third phase, which is rarely modeled in the literature;
- Preliminary results for cyclic creep tests showed good agreement when the maximum strains of each cycle are used to model the cyclic creep phenomenon. As presented, the curves have the three phases well defined, in almost total agreement with maximum strains determined experimentally.

The achieved results seem very promising for future work that may involve more varied test conditions, such as more load levels or diverse environmental conditions like temperature and humidity. Additionally, the data collected for the cyclic model may open an opportunity to better characterize this phenomenon in PSAs. In addition to the maximum strains for each cycle, it could be interesting to analyze the result for other intermediate points of the curves and thus develop a holistic approach to fully understand the phenomenon.

## Data Availability

The data presented in this study are available on request from the corresponding author. The data are not publicly available due to privacy restrictions.

## References

[1] Deshpande A., Song Z., Vaidya S. A Creep Model For Pressure Sensitive Adhesives under Shear Load. Proceedings of the 2020 Asia-Pacific International Symposium on Advanced Reliability and Maintenance Modeling (APARM).

[2] Baek S.-S., Hwang S.-H. (2016). Eco-friendly UV-curable pressure sensitive adhesives containing acryloyl derivatives of monosaccharides and their adhesive performances. Int. J. Adhes. Adhes..

[3] Huang H., Jiang Q., Dasgupta A., Mirbagheri E., Darbha K. Creep Response of Assemblies Bonded with Pressure Sensitive Adhesive (PSA). Proceedings of the InterPACK2018, ASME 2018 International Technical Conference and Exhibition on Packaging and Integration of Electronic and Photonic Microsystems.

[4] Seok W.C., Leem J.T., Song H.J. (2022). Acrylic pressure-sensitive adhesives based on ethylene glycol acrylate for flexible display application: Highly elastic and recoverable properties. Polym. Test..

[5] Silva L.F.M., Öchsner A., Adams R. (2018). Handbook of Adhesion Technology.

[6] Deng X. (2016). Progress on rubber-based pressure-sensitive adhesives. J. Adhes..

[7] Baek S.-S., Jang S.-J., Hwang S.-H. (2016). Preparation and adhesion performance of transparent acrylic pressure sensitive adhesives: Effects of substituent structure of acrylate monomer. Int. J. Adhes. Adhes..

[8] Park K.H., Lee D.Y., Yoon S.H., Kim S.H., Han M.S., Jeon S., Kim Y., Lim Y.K., Hwang D.-H., Jung S.-H. (2022). Adhesion Improvement of Solvent-Free Pressure-Sensitive Adhesives by Semi-IPN Using Polyurethanes and Acrylic Polymers. Polymers.

[9] Antosik A.K., Mozelewska K., Piątek-Hnat M., Czech Z., Bartkowiak M. (2021). Silicone pressure-sensitive adhesives with increased thermal resistance. J. Therm. Anal. Calorim..

[10] Márquez I., Paredes N., Alarcia F., Velasco J.I. (2022). Influence of polymerizable surfactants on the adhesion performance and water resistance of water-based acrylic pressure-sensitive adhesives (PSAs). J. Adhes. Sci. Technol..

[11] Fuensanta M., Martín-Martínez J.M. (2021). Structural and Viscoelastic Properties of Thermoplastic Polyurethanes Containing Mixed Soft Segments with Potential Application as Pressure Sensitive Adhesives. Polymers.

[12] Seok W.C., Park J.H., Song H.J. (2022). Effect of silane acrylate on the surface properties, adhesive performance, and rheological behavior of acrylic pressure sensitive adhesives for flexible displays. J. Ind. Eng. Chem..

[13] Lee J.-H., Shim G.-S., Park J.-W., Kim H.-J., Kim Y. (2019). Adhesion performance and recovery of acrylic pressure-sensitive adhesives thermally crosslinked with styrene–isoprene–styrene elastomer blends for flexible display applications. J. Ind. Eng. Chem..

[14] Lee J.H., Park J., Myung M.H., Baek M.-J., Kim H.-S., Lee D.W. (2020). Stretchable and recoverable acrylate-based pressure sensitive adhesives with high adhesion performance, optical clarity, and metal corrosion resistance. Chem. Eng. J..

[15] Khoramishad H., Ashofteh R.S. (2018). Influence of multi-walled carbon nanotubes on creep behavior of adhesively bonded joints subjected to elevated temperatures. J. Adhes..

[16] Tan W., Na J.-X., Zhou Z.-F. (2022). Effect of temperature and humidity on the creep and aging behavior of adhesive joints under static loads. J. Adhes..

[17] Houshyar S., Shanks R., Hodzic A. (2005). Tensile creep behaviour of polypropylene fibre reinforced polypropylene composites. Polym. Test..

[18] Wang W.-H., Huang H.-B., Du H.-H., Wang H. (2014). Effects of fiber size on short-term creep behavior of wood fiber/HDPE composites. Polym. Eng. Sci..

[19] Khabazaghdam A., Behjat B., Yazdani M., Da Silva L.F.M., Marques E.A.S., Shang X. (2020). Creep behaviour of a graphene-reinforced epoxy adhesively bonded joint: Experimental and numerical investigation. J. Adhes..

[20] Ortega-Iguña M., Chludzinski M., Sánchez-Amaya J.M. (2022). Comparative Mechanical Study of Pressure Sensitive Adhesives over Aluminium Substrates for Industrial Applications. Polymers.

[21] Eveloy V., Rodgers P., Pecht M. (2004). Reliability of Pressure-Sensitive Adhesive Tapes for Heat Sink Attachment in Air-Cooled Electronic Assemblies. IEEE Trans. Device Mater. Reliab..

[22] Townsend B.W., Ohanehi D.C., Dillard D., Austin S.R., Salmon F., Gagnon D.R. (2011). Characterizing acrylic foam pressure sensitive adhesive tapes for structural glazing applications—Part II: Creep rupture results. Int. J. Adhes. Adhes..

[23] Duan X., Yuan H., Tang W., He J., Guan X. (2021). A Phenomenological Primary–Secondary–Tertiary Creep Model for Polymer-Bonded Composite Materials. Polymers.

[24] Wu C., Wu R., Xia W., Tam L. (2019). Understanding Creep Behavior of Semicrystalline Polymer via Coarse-Grained Modeling. J. Polym. Sci. Part B Polym. Phys..

[25] Sadigh M.S., Paygozar B., da Silva L., Martínez-Pañeda E. (2020). Creep behaviour and tensile response of adhesively bonded polyethylene joints: Single-Lap and Double-Strap. Int. J. Adhes. Adhes..

[26] Boumakis I., Ninčević K., Vorel J., Wan-Wendner R. (2019). Creep rate based time-to-failure prediction of adhesive anchor systems under sustained load. Compos. Part B Eng..

[27] Geiss P., Vogt D. (2007). Durability of Pressure Sensitive Adhesive Joints.

[28] Ernault E., Diani J., Schmid Q. (2022). Single-lap joint creep behaviour of two soft adhesives. J. Adhes..

[29] Reis P.N.B., Pereira A.M., Ferreira J.A.M., Costa J.D.M. (2016). Cyclic creep response of adhesively bonded steel lap joints. J. Adhes..

[30] Li X., Qi C., Zhang P. (2019). A micro-macro confined compressive fatigue creep failure model in brittle solids. Int. J. Fatigue.

[31] Launay A., Marco Y., Maitournam M., Raoult I., Szmytka F. (2010). Cyclic behavior of short glass fiber reinforced polyamide for fatigue life prediction of automotive components. Procedia Eng..

[32] Sarkar A., Nagesha A., Parameswaran P., Sandhya R., Laha K. (2016). Insights into dynamic strain aging under cyclic creep with reference to strain burst: Some new observations and mechanisms. Part-1: Mechanistic aspects. Mater. Sci. Eng. A.

[33] Liu L., Wang X., Wu Z., Keller T. (2021). Tension-tension fatigue behavior of ductile adhesively-bonded FRP joints. Compos. Struct..

[34] Taleb L., Cailletaud G. (2011). Cyclic accumulation of the inelastic strain in the 304L SS under stress control at room temperature: Ratcheting or creep?. Int. J. Plast..

[35] Gomatam R.R., Sancaktar E. (2005). Effects of various adherend surface treatments on fatigue behavior of joints bonded with a silver-filled electronically conductive adhesive. J. Adhes. Sci. Technol..

[36] Foletti A.I.M., Cruz J.S., Vassilopoulos A.P. (2020). Fabrication and curing conditions effects on the fatigue behavior of a structural adhesive. Int. J. Fatigue.

[37] Sohn S. (2003). A new method based on application of cyclic strain to evaluate the durability of pressure sensitive adhesives. J. Adhes. Sci. Technol..

[38] Ilioni A., Badulescu C., Carrere N., Davies P., Thévenet D. (2017). A viscoelastic-viscoplastic model to describe creep and strain rate effects on the mechanical behaviour of adhesively-bonded assemblies. Int. J. Adhes. Adhes..

[39] Sadigh M.S., Paygozar B., da Silva L., Tahami F.V. (2019). Creep deformation simulation of adhesively bonded joints at different temperature levels using a modified power-law model. Polym. Test..

[40] Silva P., Valente T., Azenha M., Sena-Cruz J., Barros J. (2017). Viscoelastic response of an epoxy adhesive for construction since its early ages: Experiments and modelling. Compos. Part B Eng..

[41] Loiseau M., Chataigner S., Créac’Hcadec R., Court J.-P., Quéméré M.-O. (2021). Inverse identification method of adhesive creep properties from real scale investigations on bonded fastener. J. Adhes..

[42] Huang H., Dasgupta A., Singh N. (2021). Predictive Mechanistic Model of Creep Response of Single-Layered Pressure-Sensitive Adhesive (PSA) Joints. Materials.

[43] (2017). Testing of Rubber—Determination of Tensile Strength at Break, Tensile Stress at Yield, Elongation at Break and Stress Values in a Tensile Test.

